# Supplementary figures and images for: Relationship between fruit phenotypes and domestication in hexaploid populations of biribá (Annona mucosa) in Brazilian Amazonia
Source: PeerJ. 2023 Jan 23;11:e14659. doi: 10.7717/peerj.14659 (PMC9879159; doi:10.7717/peerj.14659)

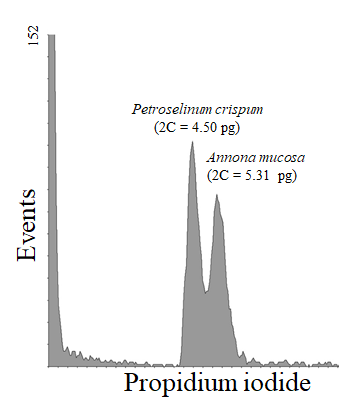

Supplement: Supplemental Information 1 — Fluorescence histograms of simultaneous analysis of propidium iodide-stained nuclei isolated from fresh tissue of internal standard Petroselinum crispum (Mill.) and Annona mucosa, respectively. [file peerj-11-14659-s001.png]

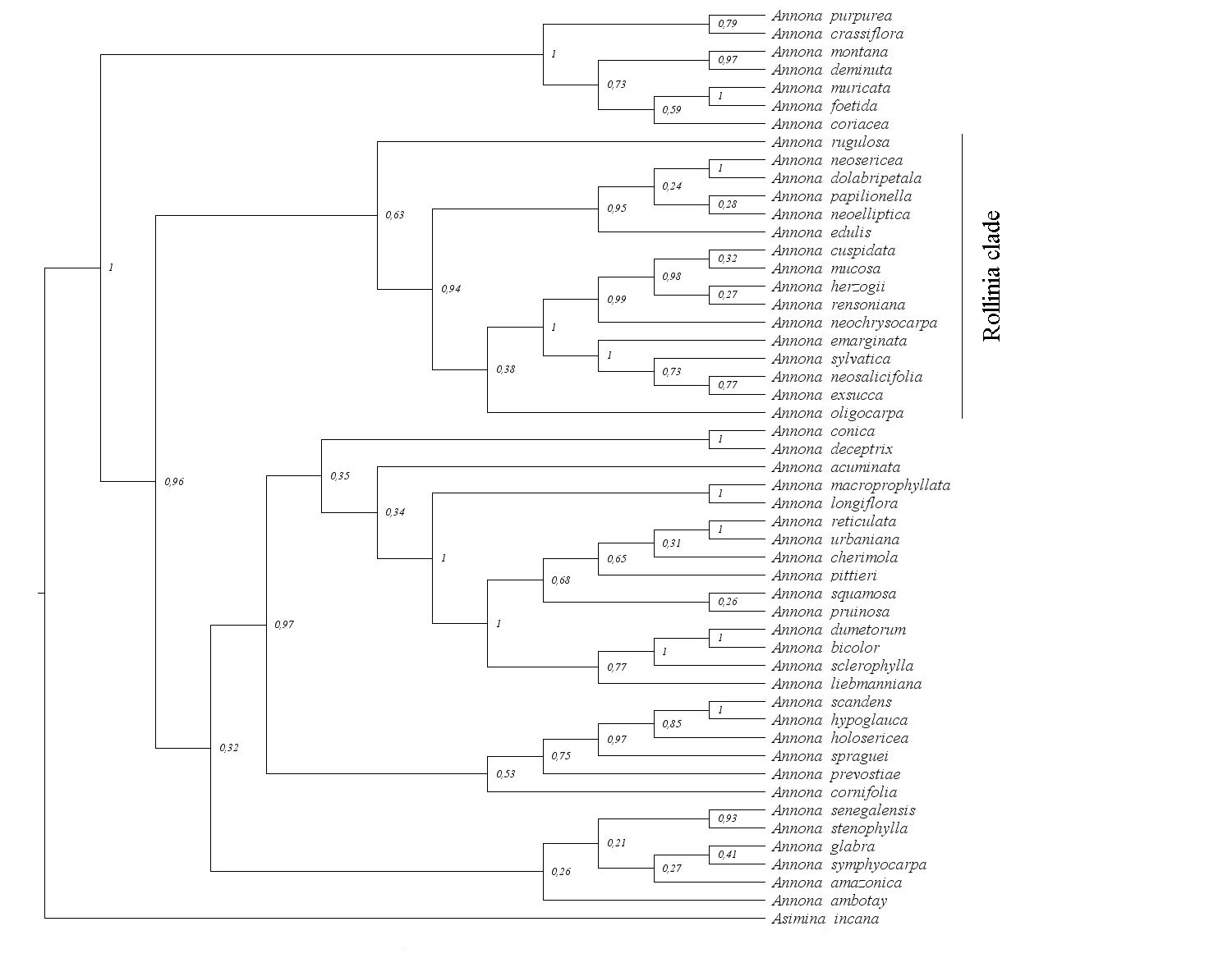

Supplement: Supplemental Information 2 — The tree was based on plastid sequences: rbcL, matK, psbA-trnH, trnL and nadH from Genebank. The number in front of the nodes represents the posterior probability of the Bayesian analysis. [file peerj-11-14659-s002.png]

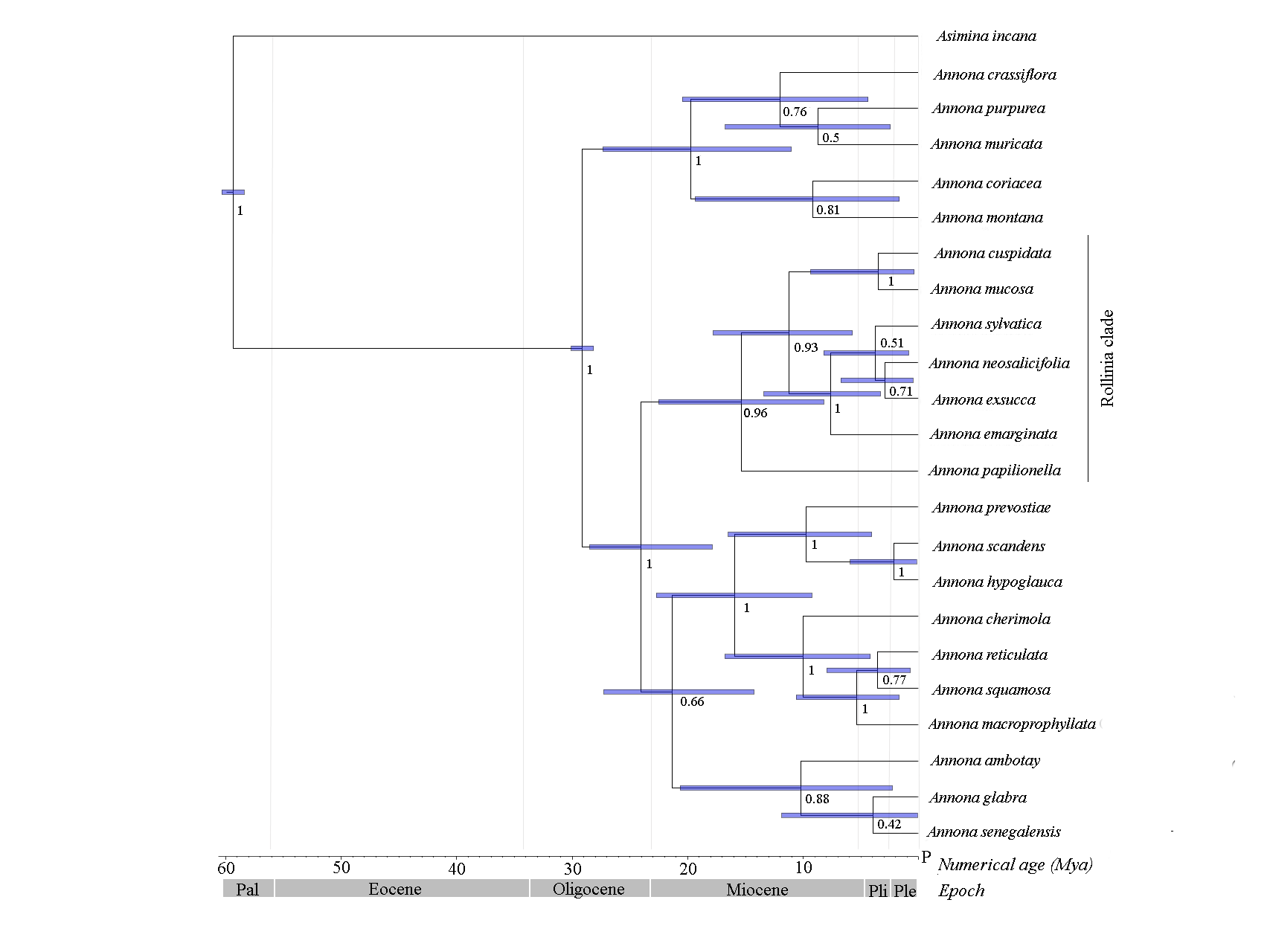

Supplement: Supplemental Information 3 — The timeline was extracted from the maximum clade credibility tree (MCC) of the BEAST analysis. Nodes represent later mean ages (Millions of years ago—Mya). The blue bars at the nodes represent the highest posterior density ranges with 95% Confidence Intervals. Numbers at nodes represent Bayesian posterior probabilities (PP). [file peerj-11-14659-s003.png]
