# Supplementary material for: Relationship between fruit phenotypes and domestication in hexaploid populations of biribá (Annona mucosa) in Brazilian Amazonia
Source: PeerJ. 2023 Jan 23;11:e14659. doi: 10.7717/peerj.14659 (PMC9879159; doi:10.7717/peerj.14659)
Supplement: Supplemental Information 4 — Missing data (-). [file peerj-11-14659-s004.docx]

**Table S1** Chromosome number, genome size and degrees of domestication available for the genus *Annona*; missing data (-).

| Species | Chromosome number | Genome size (1C) | Degree of domestication |
| --- | --- | --- | --- |
| *Annona acutiflora* Mart. | 2n = 2x = 14ª | - | - |
| *Annona ambotay* Aubl. | 2n = 2x = 14ª | - | - |
| *Annona cacans* Warm. | 2n = 2x = 14^b^ | 1^h^ | - |
| *Annona cherimola* Mill. | 2n = 2x = 14^b^ | 1.7^g^ | Domesticated ^i^ |
| *Annona coriacea* Mart. | 2n = 6x = 42^a^ | - | Wild ^j^ |
| *Annona crassiflora* Mart. | 2n = 2x = 14^f^ | - | - |
| *Annona cuspidata* (Mart.) H. Rainer* | 2n = 4x = 28^e^ | - | Incipiently  Domesticated ^i,l^ |
| *Annona dioica* A. St.-Hil. * | 2n = 2x = 14^a^ | - | - |
| *Annona emarginata* (Schltdl.) H. Rainer* | 2n = 2x = 14^c^ | 1.68^g^ | Domesticated ^i^ |
| *Annona exsucca* DC. ex Dunal* | 2n = 2x = 14^e^ 2n = 4x = 28^c^ | - | - |
| *Annona glabra* L. | 2n = 4x = 28^b^ | 1.30^h^ | Wild ^k^ |
| *Annona hispida* (Maas & Westra) H. Rainer* | 2n = 4x = 28^e^ | - | - |
| *Annona hypoglauca* Mart. | 2n = 2x = 14^c^ | - | - |
| *Annona jahnii* Saff. | 2n = 2x = 14^c^ | - | - |
| *Annona jucunda* (Diels) H. Rainer* | 2n = 2x = 14^e^ 2n = 4x = 28^c^ | - | - |
| *Annona lutescens* Saff. | 2n = 4x = 28^b^ | 1^h^ | Wild ^l^ |
| *Annona macroprophyllata* Donn. Sm. | 2n = 2x = 14^d^ | - | Domesticated ^i,k^ |
| *Annona mammifera* (Maas & Westra) H. Rainer* | 2n = 4x = 28^e^ | - | - |
| *Annona montana* Macf. | 2n = 2x = 14^b^ | - | Semi-Domesticated ^i^ |
| *Annona monticola* Mart. | 2n = 4x = 28^c^ | - | - |
| *Annona mucosa* Jacq.* | 2n = 4x = 28^e^ 2n = 6x = 42^c^ | 2.3^m^  2.7^n^  3^i^ | Domesticated ^i^ |
| *Annona muricata* L. | 2n = 2x = 14^b^ | 1.7^g^ | Domesticated ^i^ |
| *Annona neoinsignis* H. Rainer* | 2n = 2x = 14^c^ | - | - |
| *Annona neolaurifolia* H. Rainer* | 2n = 4x = 28^b^ | - | - |
| *Annona neosalicifolia* H. Rainer* | 2n = 6x = 42^g^ | 4.82^a^ | - |
| *Annona nítida* Mart. | 2n = 2x = 14^a^ | - | - |
| *Annona papilionella* (Diels) H. Rainer* | 2n = 2x = 14^e^ | - | - |
| *Annona prevostiae* H. Rainer | 2n = 2x = 14^d^ | - | - |
| *Annona purpurea* Moc. & Sesse | 2n = 2x = 14^c^ | - | Semi-Domesticated ^i,k^ |
| *Annona reticulata* L. | 2n = 2x = 14^b^ | 1.3^h^ | Semi-Domesticated ^i^ |
| *Annona salzmanni* A. DC. | 2n = 2x = 14^a^ | - | - |
| *Annona scandens* Diels. | 2n = 2x = 14^c^ | - | - |
| *Annona senegalensis* Pers. | 2n = 2x = 14^g^ | 1.7^g^ | - |
| *Annona squamosa* L. | 2n = 2x = 14^g^ | 1.2^g^ 1.63^h^ | Domesticated ^i^ |
| *Annona sylvatica* (A. St.-Hil.) Martius* | 2n = 8x = 56^c^ | - | Wild ^l^ |
| *Annona tomentosa* R. E. Fr. | 2n = 2x = 14^c^ | - | - |
| *Annona williamsii* (Rusby ex R.E.Fr.) H. Rainer* | 2n = 4x = 28^e^ | - | - |

^a^ (Morawetz 1984), ^b^ (Morawetz 1986*b*), ^c^ (Morawetz 1986*a*), ^d^ (Goldblatt & Johnson 1994), ^e^ (Maas *et al.* 1992), ^f^ (Ribeiro 2012), ^g^ (Martin *et al.* 2019), ^h^ (Leitch et al. 2019), ^i^ (Patiño 2002), ^j^ (VMABCC-BIOVERSITY 2009), ^k^ (Escobedo-López et al. 2019), ^l^ (Segura et al. 2018), ^m^ (Soares et al. 2014), ^n^ (Lorenzoni 2016)

The hyphen (-) in the domestication degrees column means lack of information, but probably represents wild.

* Asterix after author name identifies Rollinia clade species.

**References**

**Escobedo-López D, Campos-Rojas E, Rubén Rodríguez-Núñez J, Alia-Tejacal I, Núñez-Colín CA**. **2019**. Priority areas to collect germplasm of *Annona* (Annonaceae) in Mexico based on diversity and species richness indices. *Genetic Resources and Crop Evolution* **66**: 401–413.

**Goldblatt P, Johnson DE**. **1994**. *Index to plant chromosome numbers, 1990-1991*. Saint Louis: Missouri Botanical Garden.

**Leitch IJ, Johnston E, Pellicer J, Hidalgo O, Bennett MD**. **2019**. Plant DNA C-values database. *Royal Botanical Gardens, Kew*. <https://cvalues.science.kew.org/>. [accessed 14 August 2021].

**Lorenzoni RM**. **2016**. *Evidências cariotípicas e moleculares da hexaploidia em Annona mucosa.* Master's thesis, Federal University of Espírito Santo, Alegre, ES, Brazil.

**Maas PJM, Westra LYTh, Brown KSJr, Welle BJH ter, Webber AC, Thomas AL, Waha M, Heijden E van der, Bouman F, Cavé A, *et al.*** **1992**. Rollinia. *Flora, Neotropica, Monograph* **62**: 1–188.

**Martin C, Viruel M, Lora J, Hormaza JI**. **2019**. Polyploidy in fruit tree crops of the genus *Annona* (Annonaceae). *Frontiers in Plant Science* **10**: 99.

**Morawetz W**. **1984**. Karyologie, okologie und evolution der gattung *Annona* (Annonaceae) in Pernambuco, Brasilien. *Flora* **175**: 435–447.

**Morawetz W**. **1986a**. Remarks on karyological differentiation patterns in tropical woody plants. *Plant Systematics and Evolution* **152**: 49–100.

**Morawetz W**. **1986b**. Systematics and karyoevolution in Magnoliidae: Tetrameranthus as compared with other Annonaceae genera of the same chromosome number. *Plant Systematics and Evolution* **154**: 147–173.

**Patiño VMR**. **2002**. *Historia y dispersion de los frutales natives del Neotrópico.* Cali, Colombia: CIAT.

**Ribeiro, LR. 2012.** *Citogenética e efeitos citogenotóxicos de Annona crassiflora Mart. (Annonaceae)*. PhD thesis, Federal University of Lavras, Lavras, MG, Brazil.

**Segura S, Fresnedo J, Mathuriau C, López J, Andrés J, Muratalla A**. **2018**. The edible fruit species in Mexico. *Genetic Resources and Crop Evolution* **65**: 1767–1793.

**Soares JDR, Dias G de MG, Rodrigues FA, Pasqual M, Chagas EA**. **2014**. Caracterização anatômica e citométrica em biribazeiro (*Rollinia mucosa* [Jacq.]). *Revista Brasileira de Fruticultura* **36**: 272–280.

**VMABCC-BIOVERSITY. 2009**. *Libro rojo de parientes silvestres de cultivos de Bolivia.* La Paz, Bolivia: PLURAL.
