# Supplementary material for: Relationship between fruit phenotypes and domestication in hexaploid populations of biribá (Annona mucosa) in Brazilian Amazonia
Source: PeerJ. 2023 Jan 23;11:e14659. doi: 10.7717/peerj.14659 (PMC9879159; doi:10.7717/peerj.14659)
Supplement: Supplemental Information 5 — Missing data (-). [file peerj-11-14659-s005.docx]

**Table S2** Species names and GenBank accession numbers of DNA sequences used in this study. (-) missing data.

|  | Genbank access numbers | | | | |
| --- | --- | --- | --- | --- | --- |
| Species | *rbc*L | *trn*L | *mat*K | *ndh*f | *psb*A-*trn*H |
| *Annona acuminata* Saff. | GQ981664 | - | GQ981934 | - | GQ982146 |
| *Annona amazônica* R. E. Fr. | EU420853 | EU420836 | - | - | - |
| *Annona ambotay* Aubl. | *-* | - | - | - | KX248024 |
| *Annona bicolor* Urb. | EU420854 | EU420837 | - | - | - |
| *Annona cherimola* Mill. | JX571777 | KX663972 | KM068848 | KX663926 | KX664018 |
| *Annona conica* Ruiz & Pav. ex E.A. López | KX663909 | KX664000 | KX663871 | KX663955 | KX664042 |
| *Annona coriacea* Mart. | MG718393 | KP745618 | MG718904 | - | - |
| *Annona cornifolia* A. St.-Hil. | EU420855 | - | - | - | - |
| *Annona crassiflora* Mart. | MG718271 | - | MG718805 | - | - |
| *Annona cuspidata* (Mart.) H. Rainer | EU420869 | EU420851 | MH024833 | - | - |
| *Annona deceptrix* (Westra) H. Rainer | AY841595 | AY841672 | - | - | - |
| *Annona deminuta* R. E. Fr. | EU420857 | EU420839 | - | - | - |
| *Annona dolabripetala* Raddi. | MG833424 | - | - | - | - |
| *Annona dumetorum* R. E. Fr. | EU420856 | EU420838 | GQ139704 | - | GQ139844 |
| *Annona edulis* (Triana & Planch.) H. Rainer | AY841655 | AY841733 | - | - | - |
| *Annona emarginata* (Schltdl.) H. Rainer. | MG718404 | KX664004 | MG718914 | KX663961 | KX664048 |
| *Annona exsucca* DC. Ex. Dunai | - | - | - | - | KX248034 |
| *Annona foetida* Mart. | *-* | - | - | - | KX248027 |
| *Annona glabra* L. | AY841596 | AY841673 | GQ139717 | EF179281 | DQ125116 |
| *Annona* *herzogii* (R. E. Fr.) H. Rainer | AY841656 | AY841734 | DQ125062 | EF179308 | - |
| *Annona holosericea* Saff. | EU420858 | EU420840 | - | - | HG963751 |
| *Annona hypoglauca* Mart. | EU420859 | EU420841 | - | - | - |
| *Annona liebmanniana* Baill. | KX663893 | KX663982 | KX663854 | KX663936 | KX664026 |
| *Annona longiflora* S. Watson | KX663895 | KX663984 | KX663856 | KX663938 | KX664028 |
| *Annona macroprophyllata* Donn. Sm. | KM068880 | KX663980 | KM068859 | KX663934 | KX664024 |
| *Annona montana* Macfad. | EU420860 | EU420842 | KX663867 | KX663950 | KJ426607 |
| *Annona monticola* Mart. | *-* | - | - | - | - |
| *Annona mucosa* Jacq. | EU420870 | EU420852 | GQ139705 | - | GQ139845 |
| *Annona muricata* L. | AY743440 | AY743459.2 | AF543722 | EF179282 | AY841428 |
| *Annona neochrysocarpa* H. Rainer | EU420868 | EU420850 | - | - | - |
| *Annona neoelliptica* H. Rainer & Maas | JQ626090 | - | - | - | - |
| *Annona neosalicifolia* H. Rainer | KX663912 | KX664003 | KX663872 | KX663957 | KX664045 |
| *Annona neosericea* H. Rainer | MG718042 | - | MG718597 | - | - |
| *Annona oligocarpa* R. E. Fr. | EU420861 | EU420843 | - | - | - |
| *Annona papilionella* (Diels.) H. Rainer | JQ590188 | - | - | - | - |
| *Annona pittieri* Donn. Sm. | KX663898 | KX663987 | KX663858 | KX663940 | KX664029 |
| *Annona prevostiae* H. Rainer | JQ625732 | - | JQ626342 | - | KX248029 |
| *Annona pruinosa* G.E. Schatz | EU420862 | EU420844 | KX663861 | KX663943 | KX664031 |
| *Annona purpurea* Moc. & Sesse | JQ590160 | - | KM068866 | KX663945 | HG963551 |
| *Annona rensoniana* (Standl.) H. Rainer | JQ594777 | KX663994 | KX663866 | KX663949 | KX664037 |
| *Annona reticulata L.* | EU420863 | EU420845 | KM068850 | KX663929 | KX664020 |
| *Annona rugulosa* (Schltdl.) H. Rainer | JX880395 | - | JX880394 | - | - |
| *Annona scandens* Diels. | EU420864 | EU420846 | KP859349 | - | - |
| *Annona sclerophylla* Saff. | *-* | GQ139892 | GQ139718 | - | GQ139858 |
| *Annona senegalensis* Pers. | AY841597 | AY841674 | KX663870 | KX663954 | - |
| *Annona spraguei* Saff. | GQ981665 | - | GQ981935 | - | GQ982147 |
| *Annona squamosa* L. | EU420865 | KT452845 | EU715064 | KT452834 | EU715086 |
| *Annona stenophylla*  Engl. & Diels | MN166726 | - | KX146231 | - | - |
| *Annona sylvatica* (A. St.-Hil.) Martius | MG718043 | - | MG718915 | - | - |
| *Annona symphyocarpa* Sandwith | EU420866 | EU420848 | - | - | - |
| *Annona urbaniana* R. E. Fr. | EU420867 | EU420849 | - | - | - |
