# Supplementary material for: Relationship between fruit phenotypes and domestication in hexaploid populations of biribá (Annona mucosa) in Brazilian Amazonia
Source: PeerJ. 2023 Jan 23;11:e14659. doi: 10.7717/peerj.14659 (PMC9879159; doi:10.7717/peerj.14659)
Supplement: Supplemental Information 6 — For each marker, the number of species with information, the number of characters aligned in base pairs (bp), the percentage of conserved characters and the evolutionary substitution model are presented. [file peerj-11-14659-s006.docx]

**Table S3** Statistics of the markers used in the phylogenetic analysis of 50 species of Annona. For each marker, the number of species with information, the number of characters aligned in base pairs (bp), the percentage of conserved characters and the evolutionary substitution model are presented.

|  | *rbc*L | *trn*L | *mat*K | *Ndh*f | *psb*A-*trn*H |
| --- | --- | --- | --- | --- | --- |
| Number of species | 47 | 36 | 33 | 19 | 26 |
| Alignment length (sc) | 470 | 232 | 880 | 445 | 172 |
| Preserved characters (%) | 91.1 | 75.9 | 84 | 84.3 | 65.1 |
| Substitution model | K80+I | HKY+I | TPM1uF+G | HKY+I | HKY+G |
