# Supplementary material for: Relationship between fruit phenotypes and domestication in hexaploid populations of biribá (Annona mucosa) in Brazilian Amazonia
Source: PeerJ. 2023 Jan 23;11:e14659. doi: 10.7717/peerj.14659 (PMC9879159; doi:10.7717/peerj.14659)
Supplement: Supplemental Information 7 [file peerj-11-14659-s007.docx]

**Table S4** Log-likelihood and Akaike information criterion (AIC) score estimates for the dataset analyzed by the ChromEvol.

| Model | Log-likelihood | AIC |
| --- | --- | --- |
| CONST_RATE | -57.43 | 120.9 |
| CONST_RATE_DEMI | -32.8 | 71.6 |
| CONST_RATE_DEMI_EST | -32.6 | 73.2 |
| CONST_RATE_NO_DUPL | -74.17 | 152.3 |
| LINEAR_RATE | -65.5 | 141 |
| LINEAR_RATE_DEMI | -66.35 | 142.7 |
| LINEAR_RATE_DEMI_EST | -65.91 | 143.8 |
| LINEAR_RATE_NO_DUPL | -68.46 | 144.9 |
| BASE_NUMBER | -29.84 | 69.67 |
| BASE_NUMBER_NO_DUPL | -29.96 | 67.93 |
